# RUNX‐mediated growth arrest and senescence are attenuated by diverse mechanisms in cells expressing RUNX1 fusion oncoproteins

**DOI:** 10.1002/jcb.26443

**Published:** 2017-11-20

**Authors:** Gail Anderson, Nancy Mackay, Kathryn Gilroy, Jodie Hay, Gillian Borland, Alma McDonald, Margaret Bell, Siti Ayuni Hassanudin, Ewan Cameron, James C. Neil, Anna Kilbey

**Affiliations:** ^1^ Molecular Oncology Laboratory Centre for Virus Research, Institute of Infection, Immunity and Inflammation, University of Glasgow Glasgow UK; ^2^ School of Veterinary Medicine University of Glasgow Glasgow UK

**Keywords:** leukemogenesis, RUNX1‐ETO, senescence, TEL‐RUNX1

## Abstract

RUNX gene over‐expression inhibits growth of primary cells but transforms cells with tumor suppressor defects, consistent with reported associations with tumor progression. In contrast, chromosomal translocations involving *RUNX1* are detectable in utero, suggesting an initiating role in leukemias. How do cells expressing RUNX1 fusion oncoproteins evade RUNX‐mediated growth suppression? Previous studies showed that the TEL‐RUNX1 fusion from t(12;21) B‐ALLs is unable to induce senescence‐like growth arrest (SLGA) in primary fibroblasts while potent activity is displayed by the RUNX1‐ETO fusion found in t(8;21) AMLs. We now show that SLGA potential is suppressed in TEL‐RUNX1 but reactivated by deletion of the TEL HLH domain or mutation of a key residue (K99R). Attenuation of SLGA activity is also a feature of RUNX1‐ETO9a, a minor product of t(8;21) translocations with increased leukemogenicity. Finally, while RUNX1‐ETO induces SLGA it also drives a potent senescence‐associated secretory phenotype (SASP), and promotes the immortalization of rare cells that escape SLGA. Moreover, the RUNX1‐ETO SASP is not strictly linked to growth arrest as it is largely suppressed by RUNX1 and partially activated by RUNX1‐ETO9a. These findings underline the heterogeneous nature of premature senescence and the multiple mechanisms by which this failsafe process is subverted in cells expressing RUNX1 oncoproteins.

## 1 INTRODUCTION

The phenomenon of oncogene‐induced‐senescence (OIS) was first described in primary murine fibroblasts expressing oncogenic Ras^1^ and despite early scepticism was firmly established as a physiologically relevant failsafe mechanism that protects against cancer development.^2^, ^3^, ^4^, ^5^ More recently it has become clear that oncogene‐induced senescence is just one facet of cellular senescence that may result from a variety of stimuli that include DNA damage, tumor suppressor activation and epigenetic perturbation. While the positive outcomes of cellular senescence include tumor suppression and tissue repair, it may also lead to age‐related degeneration and tumor progression (reviewed in ref.^6^). The latter response appears to be associated with the senescence‐associated secretory phenotype (SASP) a phenomenon that results from release of an array of cytokines and other agonists with proinflammatory and/or pro‐oncogenic activities.^7^

Primary fibroblasts have provided an informative system to study the role of individual genetic lesions in these processes, as unlike cancer cell lines these cells are at an early developmental stage, retain normal karyotypes and display a finite lifespan.^8^ Over‐expression of any of the three *RUNX* genes induces a potent senescence‐like growth arrest (SLGA) in primary fibroblasts but by a more immediate mechanism than Ras OIS, which manifests as a response to hyper‐proliferation and DNA damage signaling.^9^, ^10^ The wider relevance of these observations in primary fibroblasts is underlined by the growth suppressive effects of *RUNX* in human CD34^+^ cells and murine stem and progenitor cells, B cells and foetal thymocytes.^11^, ^12^, ^13^ Crucially, primary fibroblasts lacking functional Arf/p53 fail to undergo RUNX SLGA and become tumorigenic,^9^ recapitulating the in vivo collaboration of Runx over‐expression and p53 deficiency in lymphomagenesis^14^ and illuminating the action of RUNX genes as “conditional oncogenes” that require collaborating genes to reveal their latent oncogenic potential.^15^ Moreover, RUNX functions appear to be necessary for Ras OIS, as indicated by the failure of senescence and oncogenic transformation of Runx2‐deficient murine fibroblasts.^10^

*RUNX1* is one of the most frequently involved genes in human leukemia where it is subject to a range of chromosomal translocations, loss of function mutations and copy number gains, while all three murine *Runx* genes act as targets for transcriptional activation by insertional mutagenesis in lymphoma models, highlighting the dualistic potential of RUNX factors to act as oncogenes or tumor suppressors according to context.^16^ The archetypal chromosomal fusions involving RUNX1 are the t(8;21) translocation which results in C‐terminal truncation of RUNX1 and fusion to ETO in acute myeloid leukemia and the t(12;21) translocation which fuses an almost complete RUNX1 isoform at its N‐terminus to a truncated TEL/ETV6 moiety in childhood B‐ALL.^17^ Notably, these translocations appear as early events in leukemogenesis that often arise in utero, as indicated by their detection in neonatal blood spots.^18^, ^19^ Latency periods to detectable disease can be protracted, supporting the existence of long lived or stable parental clones requiring collaborating secondary mutations for leukemic progression.^18^, ^20^ Further evidence that RUNX1 is not a typical tumor suppressor is provided by the observations that leukemia cells require normal RUNX1 expressed from the unaffected allele for viability,^21^ while progressing t(12;21) leukemias show sustained high level expression of RUNX1 and frequent copy number gains of chromosome 21.^22^, ^23^

The consequences of oncogenic fusions for SLGA potential are enigmatic, as the TEL‐RUNX1 (TR) fusion appears to have lost this activity despite retention of an almost full‐length RUNX1 moiety, while the RUNX1‐ETO fusion (RE) that carries a C‐terminally truncated RUNX1, induces intense SLGA in primary fibroblasts and haematopoietic progenitor cells.^24^, ^25^ However, SLGA induced by RUNX1 and RUNX1‐ETO are mechanistically distinct, as they display distinct morphological features and while both require intact p53, only RUNX1‐ETO is able to induce SLGA in p16^CDKNA2^ deficient fibroblasts.^24^ In this study we show that attenuation of senescence activity is also a feature of RUNX1‐ETO9a, a splice variant of RUNX1‐ETO with markedly increased leukemogenicity in mouse models.^26^ The paradoxical strong induction of SLGA by RUNX1‐ETO appears to be counterbalanced by a prolific SASP response and an ability to promote immortalization and outgrowth of cells that escape from SLGA. Our findings demonstrate multiple mechanisms by which transformed cells escape from RUNX growth suppression and provide a rationale for the contrasting secondary collaborating mutations required for TEL‐RUNX1 and RUNX1‐ETO associated leukemias.

## 2 MATERIALS AND METHODS

### 2.1 Cells and viral vectors

Hs68 human foreskin fibroblasts (Sigma‐Aldrich, Gillingham,UK), primary murine embryonic fibroblasts (MEFs—prepared in house^9^) and 293T cells (ATCC) were maintained in DMEM (Invitrogen, Paisley, UK) supplemented with 10% foetal calf serum (FCS), 2 mM l‐glutamine and 100 units each of penicillin and streptomycin. REH lymphocytic leukaemia cells (ATCC) and EBV‐transformed lymphoblastoid cell line, LCL114 (a kind gift from Professor Ruth Jarrett) were maintained in RPMI 1640 (Invitrogen) supplemented as above. Lentiviral vectors were based on the pLenti6 plasmid (Addgene, Teddington, UK) carrying the puromycin selectable marker. The RUNX1 constructs contain a 1.6 kb EcoR1 fragment encoding either RUNX1P1 or P2; P1 is the control isoform for TR experiments, P2 for those comparing RE/RE9a. RE is a 2.2 kb Xba1 fragment and TR is a 2.5 kb EcoR1 fragment all excised from the corresponding pBabe‐PURO retroviral construct^24^ and subcloned into the polylinker region of pLenti‐PURO. RE9a is a 1.9 kb fragment amplified from MIPAE9a (A kind gift from Professor D.E. Zhang, The Scripps Research Institute, La Jolla, Ca.) using EcoR1 tagged forward (5′‐CGAGAATTCTCGAGGTTGATCTCTCGAGG) and reverse (5′‐ CGGAATTCGTTAATTCACTAGTGATTCCATCG) primers and subcloned as above. TRΔHLH was excised on an EcoR1‐Not1 fragment from pMSCV TRΔHLH‐IRES‐GFP (a kind gift from Prof. O. Williams, UCL, London). The overhanging 5′ ends (Not1/pMSCV and Sal1/pBabe‐PURO) were blunt ended using a Klenow Fill‐in Kit (Agilent Technolgies, Stockport, UK) according to the manufacturer's protocol before sequential EcoR1 digestion and ligation. TRK99R was generated from pBabeTR^24^ by site‐directed mutagenesis using the QuikChange II Site‐Directed Mutagenesis Kit (Agilent Technologies) according to the manufacturer's instructions. The mutant strand synthesis reaction was performed on 5‐50 ng template DNA and 125pMol of primer pairs TRK99Rf (5′‐CTGCTGCTGACCAGAGAGGACTTTCGC) AND TRK99Rr (5′‐GCGAAAGTCCTCTCTGGTCAGCAGCAG‐).

### 2.2 Lentivirus transduction

293T cells were plated at 1.1 × 10^7^ in T150 flasks and incubated overnight at 37°C. Transfections were carried out according to the manufacturer's instructions for adherent cells using Lipofectamine 2000 (Invitrogen). 8 μg psPAX2 and 5 μg pCMV‐VSVG helper plasmids (kindly gifted by Dr J. van Tuyn, Beatson Institute, Glasgow) were transfected with 20 μg of each pLenti‐PURO construct. Viral supernatants were harvested after 48 and 72 h incubations at 37°C, filtered through 0.45 μm filters (Sartorius, Epsom, UK) and concentrated by ultra centifugation for 2 h at 166 880g. Supernatants were frozen in aliquots at −80°C. Hs68 fibroblasts were plated at 8 × 10^5^ in 10 cm dishes and incubated overnight before infection at a multiplicity of infection (MOI) of 1 for 12‐16 h in the presence of 4 μg/mL polybrene (Sigma). Selection was performed using 2 μg/mL puromycin for 4 days. Control uninfected Hs68 fibroblasts died under these selection conditions. Following selection Hs68s were removed from the culture dish, counted for viable cells and used for experimental analysis.

### 2.3 Lentiviral titration

NIH 3T3 cells were seeded at 8 × 10^3^ per well in 12‐well plates, incubated overnight, then the cells were fed with 1 mL complete media supplemented with polybrene (4 µg/mL). Virus was thawed rapidly at 37°C and agitated to resuspend particles. 11 µL of virus stock were added to one well and mixed. A series of 1:5 serial dilutions were performed over six wells and plates were incubated for 48 h at 37°C. The virus/polybrene mixture was replaced with complete media supplemented with 2 µg/mL puromycin (Sigma) and incubated for 8 days. Cells were fixed with 100% methanol for 10 min then washed with PBS (pH 6.8) and stained with 10% Giemsa in PBS (pH 6.8) for 30 min. The plates were washed with water and discreet colonies counted. Plaque‐forming units (pfu)/µL was calculated.

### 2.4 Growth curves, senescence staining, and 3T3 passage culture

Cells were plated at 2.5 × 10^4^/well in 12‐well plates in selection medium containing 2 μg/mL puromycin. Live cell counts were carried out in triplicate using a haemocytometer and trypan blue as a vital stain. Media were changed every 3‐4 days. Senescence staining was assayed using a solution of X‐gal (Invitrogen) at pH 6.0 to detect SA‐β‐galactosidase activity as described.^9^ 3T3 passage culture was essentially performed according to the protocol of Todaro and Green.^27^

### 2.5 Analysis of intracellular reactive oxygen species (ROS)

To assess the generation of intracellular ROS, transduced cells were plated after puromycin selection in a six‐well dish at 3 × 10^5^ per well. After 6 days the cells were washed in PBS and then incubated in the dark for 20 mins at 37°C with 15 μM 2′, 7′‐dichlorofluorescein diacetate (DCF‐DA, Millipore, Watford, UK) before harvesting according to the manufacturer's instructions. DCF fluorescence (maximum excitation 495 nm; maximum emission 530 nm) was quantitated on an Accuri C6 flow cytometer (BD Biosciences, Oxford, UK) using a 488 nm blue laser and detecting fluorescence on the FL1 channel with a 533/30 filter. The cell population was gated on a forward scatter/side scatter plot to exclude debris. Significance values were determined by Student's *t*‐test. ROS scavenging was achieved by treating cells with sodium pyruvate (NaPyr). Sodium pyruvate was diluted in ddH2O and added to cell culture medium at 250 µM. Treatment of cells commenced on the day of antibiotic selection and medium was changed every 2‐3 days.

### 2.6 Human cytokine array

Cells were cultured at 2.5 × 10^4^ per well in 12‐well plates for 4 days before replacing the medium with low serum media (0.2% FCS) for a further 48 h. The conditioned media was harvested and diluted to control for cell number before application to a human cytokine array chip (RayBiotech, Norcross, GA). Chips were treated as specified in the manufacturer's guidelines. Chips were sent for data extraction and analysis (RayBiotech) where ODs were measured. OD values were normalised to background levels determined from the empty vector pLenti‐PURO, control cells.

### 2.7 IL‐6 ELISA

Cells were plated out at 2.5 × 10^4^ in 12‐well plates and fed with fresh media every 3 days. On day 6 post‐selection, the cell culture medium was harvested and stored at −80°C and the cells were counted. Harvested media was thawed rapidly at 37°C. A Quantikine IL‐6 ELISA (R&D Systems, Abingdon, UK) was performed in accordance with the manufacturer's protocol. Plates were analyzed at 450 nm using an ELISA plate reader. Plates were additionally read at 540 nm to remove background fluorescence. The values were normalized to cell count and final values were normalized to the empty vector control.

### 2.8 Protein extraction and Western blotting

Preparation of whole cell protein extracts was performed as described previously using 0.4% Triton,^9^ 0.5% NP40,^28^ 0.1% SDS (RIPA) or 1.0% SDS (RIPA) lysis buffer supplemented with 0.1 μg/mL okadaic acid (OA), 5 μg/mL aprotinin, 5 μg/mL leupeptin, 5 μg/mL pepstatin A, 1 mM benzamidine, and 50 μg/mL PMSF).

Samples equivalent to 20‐30 μg protein (Bio‐Rad protein concentration assay, Bio‐Rad Laboratories, Watford, UK) were resolved on SDS‐polyacrylamide gels or Novex gradient gels and electro‐blotted onto Hybond‐ECL nitrocellulose membranes (GE Healthcare, Little Chalfont, UK). Proteins were visualized using the following primary antibodies: RUNX (Caltag Medsystems, Little Balmer, UK, D207‐3), p16^INK4A^, p53, actin (Santa Cruz Biotechnology, Heidelberg, Germany, sc‐468, sc‐126, and sc‐1616), Calnexin and p19^ARF^ (Abcam, Cambridge, UK, ab22595 and ab80), p38^MAPK^ and phospho‐p38^MAPK^ (Cell Signalling Technologies, Leiden, The Netherlands, 9212 and 9211). Western blots were developed with ECL (Thermofisher Scientific, Paisley, UK) according to the manufacturer's guidelines.

### 2.9 Statistical analysis

Graphs were plotted and statistics calculated using GraphPad Prism (GraphPad Software, Inc., La Jolla, CA). Error bars relate to standard deviations. All statistical comparisons were performed using the Student's *t*‐test. Unless stated a significance value ≤0.05 is denoted by (*) ≤0.01 by (**) and ≤0.001 by (***). Growth curves and bar charts show one representative experiment of three, each performed on triplicate samples, unless otherwise stated.

## 3 RESULTS

### 3.1 Suppression of senescence‐like growth arrest by TR requires an intact HLH domain and is abolished by K99R mutation

**Figure 1:**
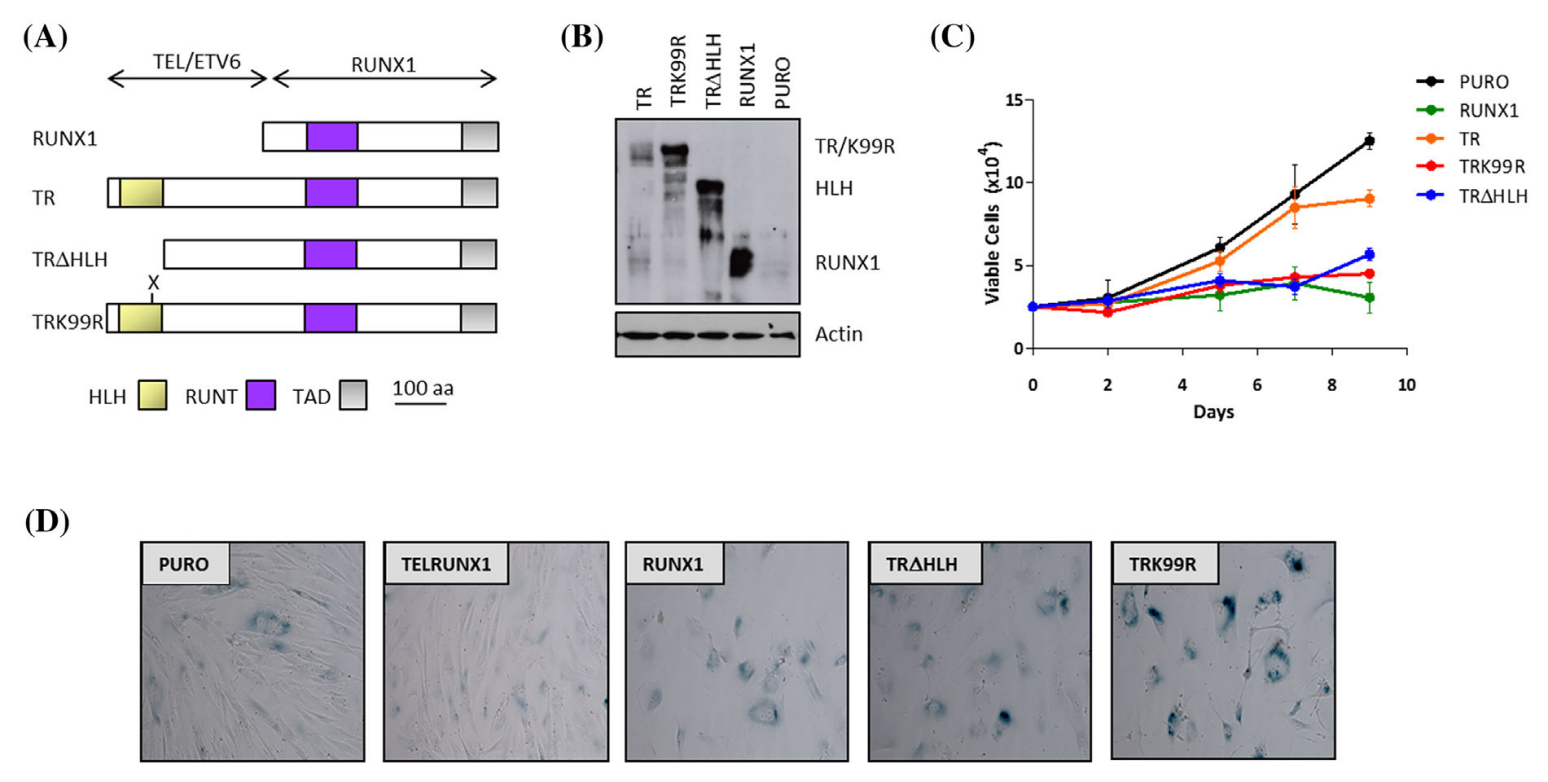
Deletion or mutation of the TEL domain reactivates SLGA activity in the TR fusion oncoprotein. Hs68 cells were transduced with lentivirus vectors encoding RUNX1, TR, TRΔHLH, TRK99R or the empty vector (PURO) control. (A) Schematic of TR and the HLH mutants, TRΔHLH & TRK99R. (B) Western blot analysis of RUNX1, TR, TRΔHLH and TRK99R expression in transduced Hs68 cells probed for RUNX. (C) Representative growth curves of transduced Hs68 cells performed over a 9 day period. (D) Images captured after staining cells for SA‐β‐Gal activity at pH6.0, 6 days post‐selection when signs of senescence were apparent

We showed previously that the TR fusion oncoprotein does not induce SLGA in primary fibroblasts despite its retention of an almost full‐length RUNX1 protein moiety.^24^ The most likely explanation for this phenomenon appeared to be a dominant suppressive effect arising from N‐terminal fusion to a TEL sequence. To test this hypothesis we examined the effect of mutating TEL functional domains in TR (Figure 1A). Previous studies showed that the TEL helix‐loop‐helix (HLH) domain, also known as the pointed domain, is required for homotypic dimerization^29^ and is also important for the leukemogenic activity of TR in haematopoietic stem cell models of B‐cell ALL.^30^ We tested the ability of a deletion mutant (TRΔHLH) to induce SLGA in Hs68 primary human fibroblasts. RUNX1 and TR were included as positive and negative controls respectively. Ectopic expression of TRΔHLH was readily detectable in fibroblasts (Figure 1B), and induced a rapid and dramatic growth arrest that was morphologically indistinguishable from RUNX1 (Figure 1C), accompanied by an intense pattern of SA‐βGal staining activity (Figure 1D). Consistent with our previous findings, Hs68 cells expressing TR retained a fibroblastic morphology and continued to divide at comparable rates to pLentiPURO control cultures. The HLH domain is required for nuclear localization of TEL, while within this domain lysine 99 has been reported as a site that is required for recruitment of the TR fusion protein to TEL nuclear bodies.^31^ We therefore tested the effect of K99R substitution on SLGA potential by introducing this mutation into our expression construct. Like the TRΔHLH mutant, TRK99R induced profound growth arrest and SA‐βgal staining (Figures 1C and 1D).

**Figure 2:**
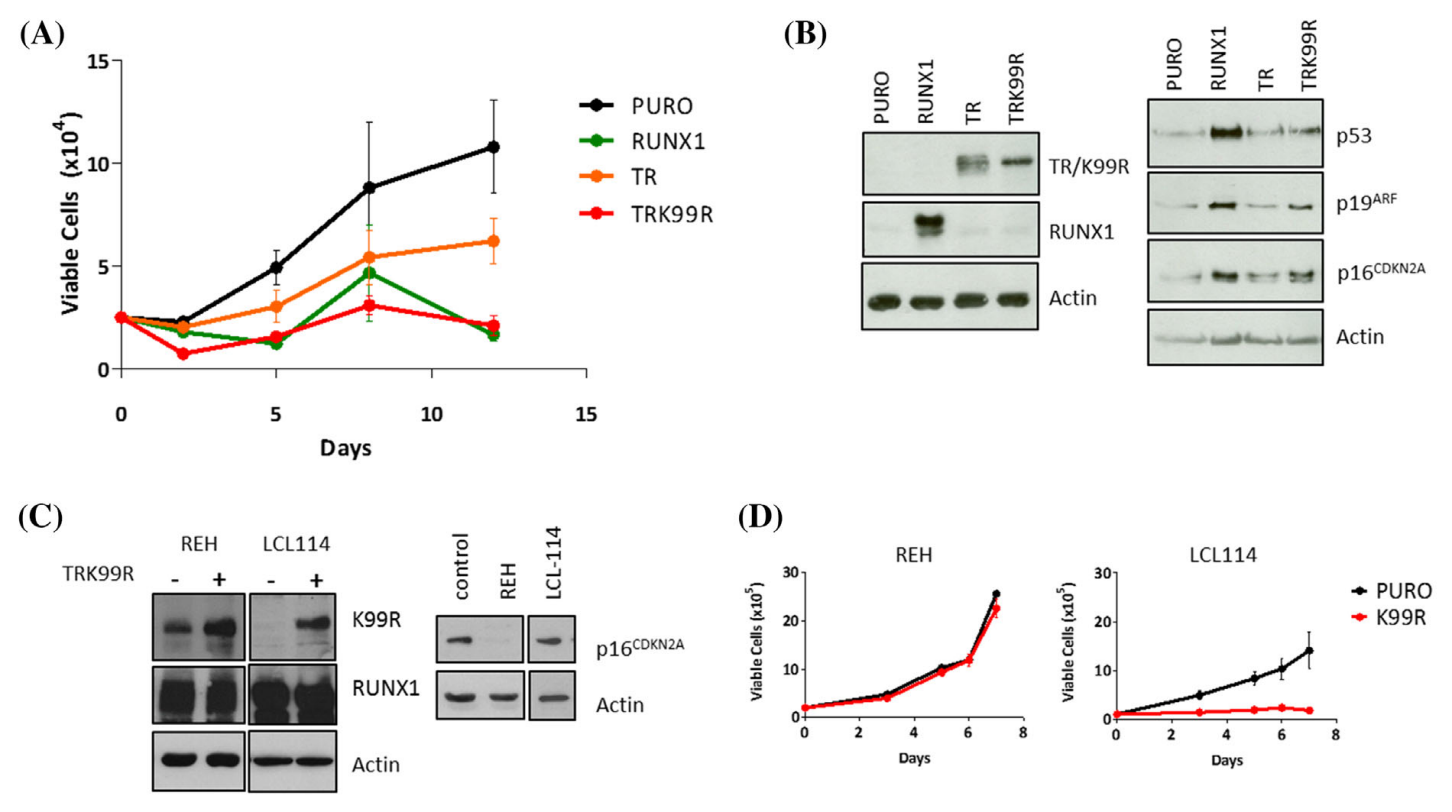
Reactivation of failsafe pathways induced by the mutated TR fusion protein reveals roles of p53 and p16^CDKN2A^ in RUNX1 SLGA. Primary MEFs were transduced with retroviral vectors encoding RUNX1, TR, TRK99R or the empty vector (PURO) control. (A) Growth curves on MEF cells expressing RUNX1, TR, TRK99R or Puro empty vector control. Mean viable cell counts were plotted over the course of 12 days. (B) Western blot analysis showing relative levels of p19, p16^CDKN2A^ and p53 in response to TR at d13. (C left panel) EBV‐immortalised human B cells (LCL114) and REH cells were transduced with lentiviral vectors expressing TRK99R (+) or the pLenti‐Puro empty vector control (−). TRK99R expression levels analysed by western blotting in both cell types. (C right panel) Western blot analysis of p16^INK4A^ expression levels in the LCL114 and REH cells. (C) Representative growth curves of the LCL114 and REH transduced with TRK99R or Puro over 7 days

Similar results were observed in primary murine embryonic fibroblasts (MEFs) where RUNX1 and TRK99R induced a profound growth arrest while TR cells grew, albeit more slowly than vector transduced control cells (Figure 2A). Analysis of senescence‐associated markers in these cells (Figure 2B) showed that the K99R mutation reactivates the induction of p19Arf/p53 signalling as well as p16^*CDKN2A*^. By contrast, TR had no effect on these senescence markers indicating that it acts neither as an inducer nor as a dominant negative repressor of these failsafe responses.

### 3.2 End stage leukemia cells are refractory to growth arrest by TRK99R

RUNX1 strongly induces expression of p16^CDKN2A^ and our previous studies showed that RUNX1 SLGA fails in Leiden human fibroblasts that lack expression of p16^CDKN2A^ due to a homozygous mutation that does not affect the p14^ARF^ reading frame.^24^, ^32^ Notably, p16^CDKN2A^ expression can also be lost in t(12;21) leukemias due to gene deletion^33^ or promoter hypermethylation in ALL^34^ and gene deletion has been detected in the prototypic REH cell line.^35^ It was therefore interesting to test the resistance of REH cells to ectopic RUNX1. However, high basal levels of RUNX1 expression obscure measurement of ectopic RUNX1 expression in these cells. For this reason we used the more readily detectable TRK99R as a surrogate inducer of SLGA. As shown in Figure 2C, ectopic expression of TRK99R was detectable by Western blot analysis of REH cells and an EBV‐immortalized lymphoblastoid cell line which served as a control line with intact p16^CDKN2A^ expression. Notably, only the LCL line was susceptible to growth arrest while REH tolerated TRK99R expression without any obvious phenotypic effect (Figure 2D).

### 3.3 Senescence‐like‐growth‐arrest is attenuated in RE9a, a leukemogenic variant isoform of RUNX1‐ETO

**Figure 3:**
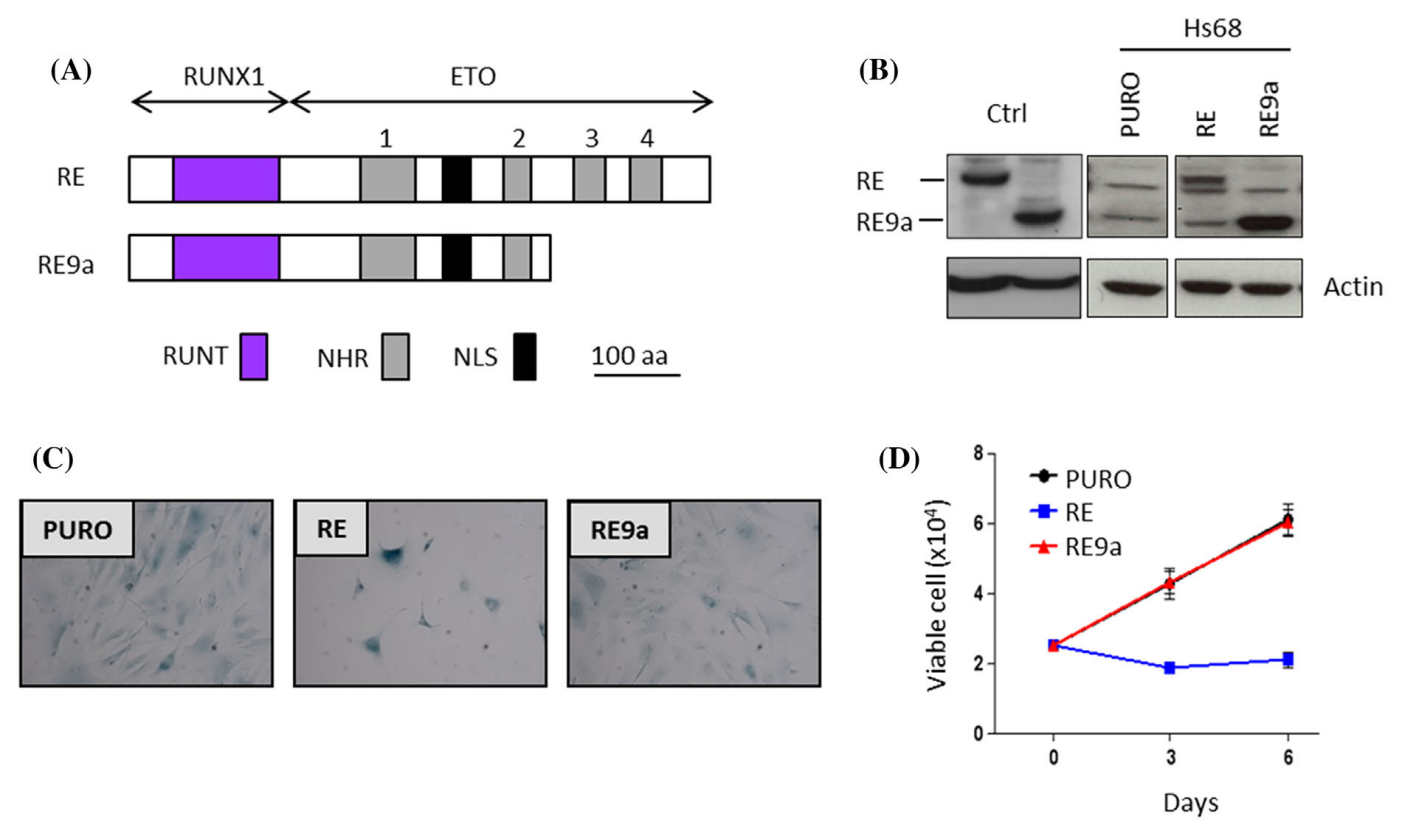
RE9a displays markedly attenuated SLGA in primary human fibroblasts. Hs68 cells were transduced with lentiviral vectors encoding RE, RE9a or the empty vector (PURO) control. (A) Schematic of the RE9a spliced variant compared to RE. (B) Western blot analysis of RE and RE9a expression in transduced Hs68 cells probed for RUNX. (C) Images captured after staining cells for SA‐β‐Gal activity at pH6.0, 6 days post‐selection when signs of senescence were apparent. (D) Representative growth curves of transduced Hs68 cells performed over a 6 day period

In contrast to TR, the RUNX1‐ETO (RE) fusion which is found in many cases of AML induces a potent SLGA in primary human foreskin fibroblasts.^24^ However, the t(8;21) fusion also generates a minor spliced variant of RE lacking two C‐terminal ETO repressor domains that is much more potently leukemogenic in mouse models^26^ (Figure 3A). To determine whether the increased leukemogenic potential of RE9a is associated with attenuation of SLGA, we introduced RE and RE9a into primary human foreskin fibroblasts (Hs68) and examined the cells for markers of this anti‐cancer failsafe mechanism. Ectopic expression levels of RE9a were greater than for RE (Figure 3B) but failed to induce a flattened morphology or perinuclear SA‐βGal staining typical of RE‐expressing cells (Figure 3C). Moreover the Hs68 cultures continued to proliferate with comparable kinetics to the vector controls indicating that cell growth was refractory to RE9a expression (Figure 3D).

**Figure 4:**
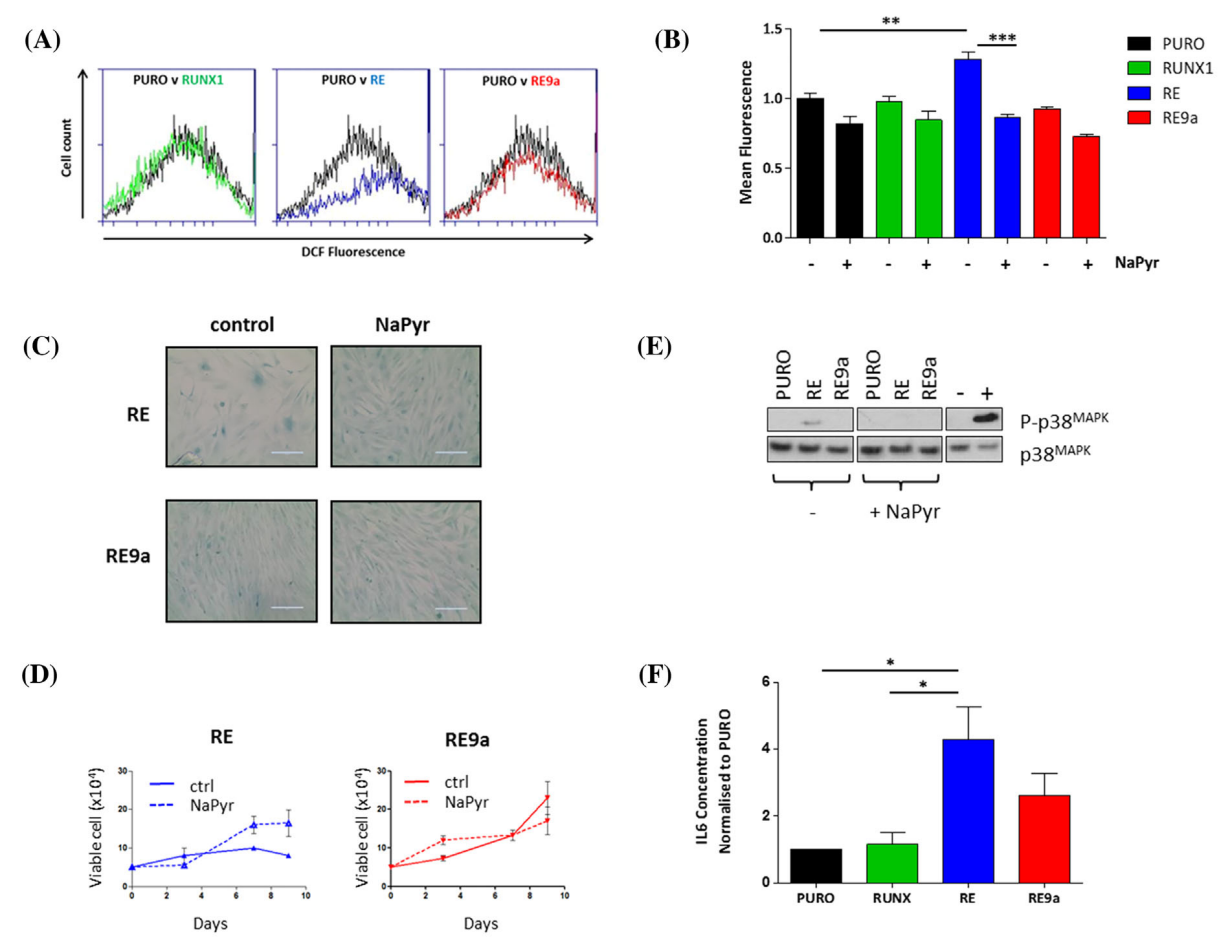
Attenuation of ROS production, MAPK signalling and IL‐6 production in RE9a‐expressing human fibroblasts compared to RE. Hs68 cells were transduced with lentiviral vectors encoding RE, RE9a or the empty vector (PURO) control and treated at the point of selection with NaPyr. (A) Intracellular ROS levels in response to NaPyr analyzed 6 days after puromycin selection by flow cytometry‐based detection of DCF‐DA fluorescence. The data shown are representative of three independent biological replicates. (B) Mean fluorescence for the experiment shown in A was quantified and normalized to the empty vector (PURO) control and the data plotted as a bar chart. (C) Images captured after staining cells for SA‐β‐Gal activity at pH6.0 on the indicated cell populations grown in the presence and absence of NaPyr for 6 days when signs of senescence were apparent. (D) Representative growth curves of the same cell populations performed over a 10 day period under the same conditions. (E) Western blot analysis of phospho‐p38MAPK, p38MAPK, and p16^CDKN2A^ expression in the indicated cell populations grown in the presence and absence of NaPyr for 6 days. (F) Supernatant collected from cells 6 days post‐selection was subjected to ELISA for IL‐6. Bar chart shows fold change in IL‐6 concentration ± SD compared with PURO control

As reported, RUNX1‐ETO SLGA is associated with accumulation of intracellular ROS and p38MAPK phosphorylation in primary human fibroblasts.^24^ To assess the capacity of RE9a to induce these responses we transduced Hs68 fibroblasts with RE, RE9a, and the empty vector control (pLenti‐puro) and treated at the point of puromycin selection with sodium pyruvate to scavenge for intracellular ROS. Six days after re‐plating when visible signs of senescence were apparent, ROS were quantitated by FACS‐based detection of emissions from the peroxide‐sensitive fluorophore DCF‐DA. As shown in Figure 4A, RE was confirmed as an inducer of intracellular ROS which failed to accumulate above background levels in response to RUNX1 or RE9a. Moreover scavenging in the presence of sodium pyruvate reduced detectable ROS for all cultures but was most striking for RE where background levels of ROS were restored (Figure 4B). Restoration of ROS levels was accompanied by morphological reversion and normal proliferation for RE, but had no effect on RE9a cultures (Figures 4C and 4D).

To determine the consequences of successful scavenging for p38MAPK activation, we analyzed expression levels of phospho‐p38MAPK by Western blotting in the presence and absence of sodium pyruvate. As shown in Figure 4E induction of phospho‐p38MAPK was unique to RE‐expressing cells and completely abolished in the presence of sodium pyruvate. Together these data indicate a requirement for intracellular ROS for activation of p38MAPK and SLGA in response to RE. Moreover it appears likely that the failure of RE9a to induce SLGA reflects loss of function or reduced engagement of ROS/p38MAPK signalling pathways.

The induction by RE of ROS, a potentially tumor promoting response^36^, suggested to us that the unique SLGA associated with RE might have pro‐oncogenic features. We therefore examined senescent cells for release of IL‐6, a major component of the senescence‐associated secretory phenotype.^6^ As shown in Figure 4F, supernatant from Hs68 cells transduced with RUNX1, RE, or RE9a were tested for the presence of IL‐6 six days after selection by ELISA. This analysis showed greater than fourfold increase in IL‐6 release from RE‐expressing cells. RUNX1‐transduced cells showed no significant increase, while RE9a cells showed a partial response, despite the lack of growth arrest and morphological changes associated with SLGA in these cells.

### 3.4 RE induces a uniquely potent senescence‐associated secretory phenotype

**Figure 5:**
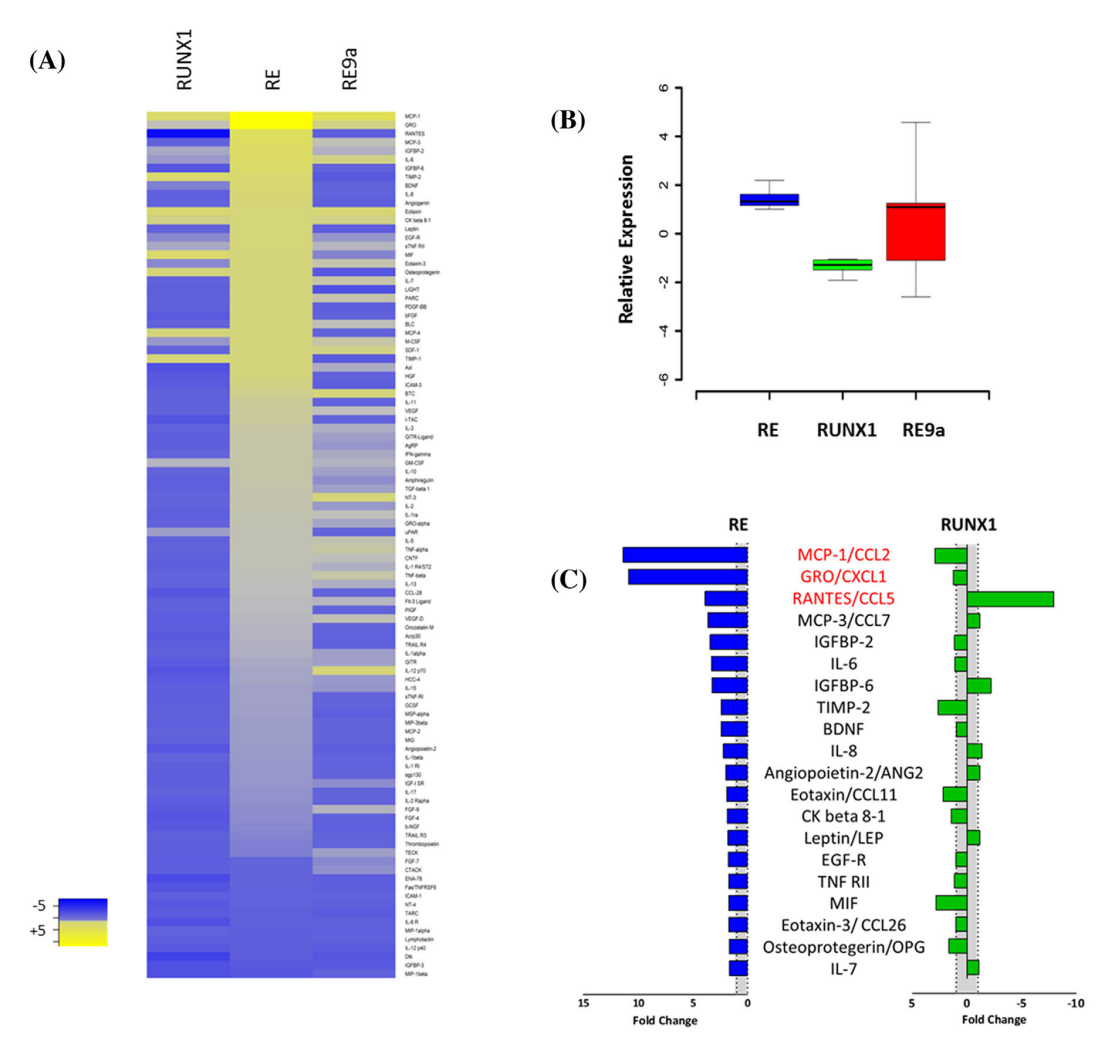
RE induces a uniquely potent SASP in human fibroblasts. (A) Supernatant collected from transduced Hs68 cells 6 days post selection was tested on a human cytokine array to measure SASP induction by RUNX1, RE, and RE9a. Yellow indicates up‐regulation relative to the PURO control while blue indicates down‐regulation, while gray is neutral. (B) The relative levels of all SASP components released by cells expressing RUNX1, RE, and RE9a are depicted as box‐whisker plots. The boxplot shows the distribution of cytokine expression, with the box representing the 1st to 3rd quantiles (Q1‐Q3) and the midline representing the median. Whiskers represent either the most extreme data point or 1.5× the Q1‐Q3 interquartile range, whichever is smaller. (C) The fold change in expression in RE (blue bars) and RUNX1 (green bars) of the 20 SASP components most up‐regulated in RE (relative to the Puro control). The gray shaded area depicts the baseline. Red text indicates selected cytokines with a pro‐oncogenic effect

Our findings suggested that the RE‐associated SASP is at least partly dissociable from growth arrest as IL‐6 is not induced in senescent cells expressing RUNX1, but is significantly induced by RE9a expressing Hs68 cells which display normal morphology and growth rates. To test this hypothesis further we analyzed conditioned media using arrays containing antibodies against 120 secreted proteins (Raybiotech). OD values were adjusted to exclude background fluorescence and normalized to the empty vector pLenti‐puro control. The full dataset is listed in Table S1. For presentation in Figure 5A, the top 100 altered SASP components were sorted on the values obtained from the RE array, with yellow indicating up‐regulation, blue down‐regulation, and gray the basal levels of secretion (equivalent to control). Conditioned media from RE‐expressing cells displayed a robust SASP with the majority of secretory markers up‐regulated relative to the empty vector control (*P* = 7.4 E‐6; students *t*‐test). In contrast RUNX1 displayed a largely repressive profile (*P* = 5.4 E‐30) suggesting that senescence secretion is not a major contributor to RUNX1‐induced SLGA. RE9a generated an intermediate SASP profile despite the normal growth appearance of Hs68 cells expressing RE9a. As further illustrations of the contrasting effects of RE and RUNX1, the box‐whisker plot in Figure 5B shows the most significantly changed SASP components for RE (*n* = 87), which are largely repressed by RUNX1, and display heterogeneous responses to RE9a. Also, the most strongly up‐regulated exemplars from the RE SASP are shown in Figure 5C where again it is clear that most are either repressed or minimally induced by RUNX1. Notably, the top 3 RE SASP components (in red typeset) are cytokines with annotated pro‐oncogenic functions (CCL5, CCL2, and CXCL1).^37^, ^38^, ^39^ These observations suggest that the RE and RUNX1 SASP responses are likely to have divergent paracrine effects on bystander cells.

### 3.5 RE but not RUNX1 allows escape from senescence and promotes immortalization of primary murine fibroblasts

**Figure 6:**
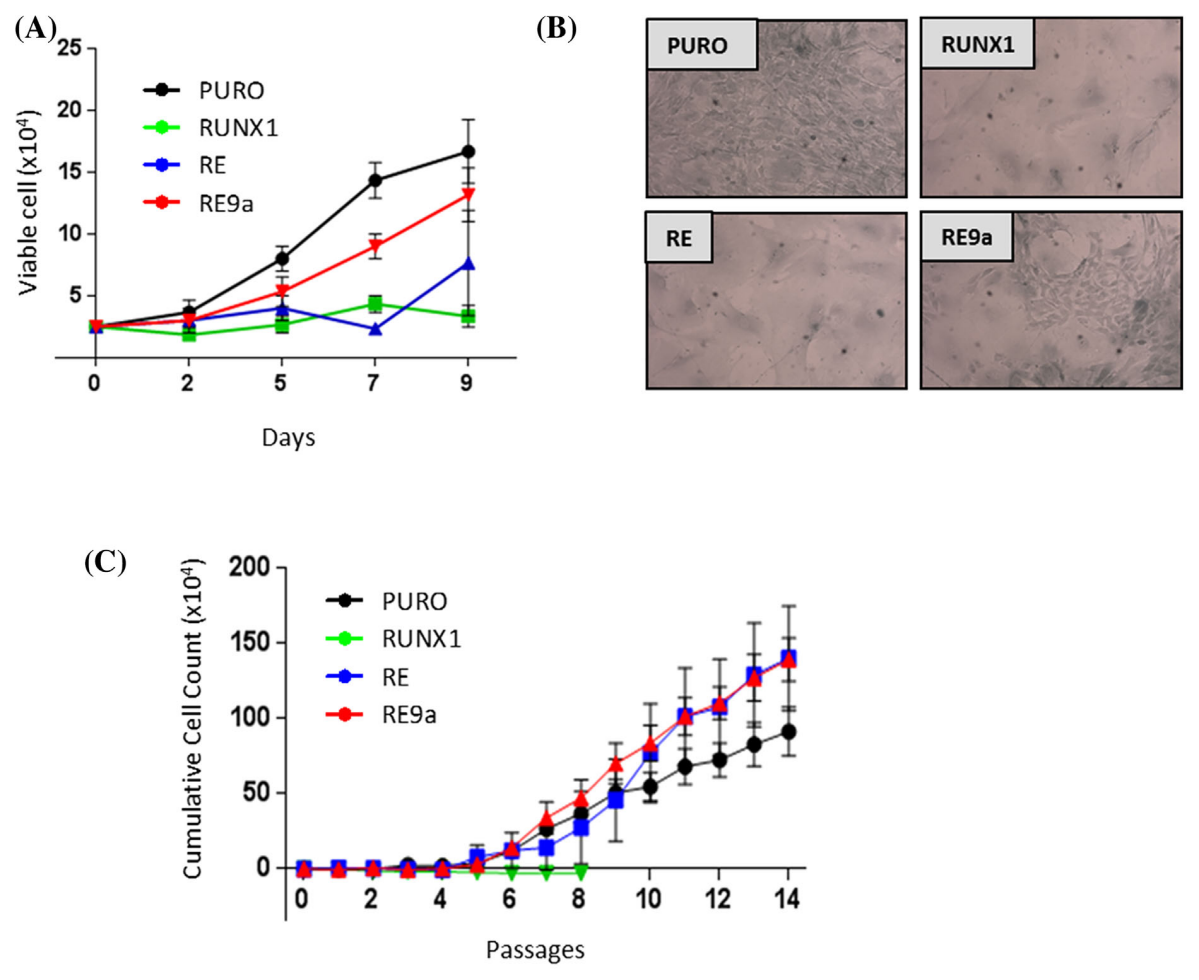
RE and RE9a but not RUNX1 promote the immortalization and outgrowth of primary murine fibroblasts. Primary MEFs were transduced with retroviral vectors encoding RUNX1, RE, RE9a, or the empty vector (PURO) control. (A) Representative growth curves of transduced cells performed over a 9 day period. (B) Images captured after staining cells for SA‐β‐Gal activity at pH6.0, 6 days post‐selection when signs of senescence were apparent. (C) 3T3 passage cultures on four independent primary MEF cultures expressing RUNX1, RE or RE9a. Mean cumulative cell counts were plotted over the course of 14 passages. RUNX1 cultures could not be split beyond passage 8 due to senescence

The finding that RE induces a robust and potentially pro‐oncogenic SASP led us to consider whether its potent growth suppression might be reversed in some circumstances. We examined prolonged expression of RE in primary MEFs which are prone to early growth arrest under normoxic conditions but can proliferate and often undergo spontaneous immortalization when grown upon 3T3 passage culture^27^ in low oxygen tension. As previously reported^9^, ^24^ introduction of RE or RUNX1 into primary MEFs was accompanied by a profound growth arrest (Figure 6A) and flattened morphology (Figure 6B) typical of senescent cells. RE9a displayed an attenuated senescent phenotype in these cells, with some areas of flattened cells but also foci of fibroblastic cells that rapidly expanded within the cultures (Figures 6A and 6B). Consistent with their persistent growth, cells expressing RE9a underwent spontaneous immortalization after approximately 6‐7 passages (Figure 6C). More surprising was the spontaneous but slightly delayed immortalization of MEFs expressing RE after approximately 8‐9 passages. Rapid outgrowth was observed in 4 out of 4 MEF cultures, which overtook vector control cells in cumulative cell counts. In contrast, RUNX1‐expressing cells failed to expand with serial passage. These findings further illustrate the contrast between SLGA induced by RUNX1 and RE and suggest a link between the SASP and the ability of RE cells to escape from senescence.

## 4 DISCUSSION

This study elucidates the ability of cells expressing RUNX1 fusion oncoproteins to evade RUNX growth suppressive potential and supports the predictive value of insights from primary cell systems for the in vivo behavior of mutant cancer genes. In two out of three cases (TR, RE9a), RUNX1 SLGA activity is lost or markedly attenuated in the oncoprotein derivates, while in the case of RE it appears to be compensated by a pro‐oncogenic secretory response that is associated with escape from senescence and immortalization.

We have shown that RUNX1 SLGA is masked in the TR oncoprotein by fusion to the TEL domain and can be reactivated by deletion of the TEL HLH domain or by a single mutation in the HLH domain of lysine 99 to arginine. Notably, this mutation has been reported to induce a shift in protein localization and affects a reported site of post‐translational modification. Sumoylation of the corresponding site in TEL has been observed,^31^, ^40^ although there are multiple alternative post‐translational modifiers that target exposed lysine residues that may also be relevant.^41^ Unequivocal identification of enzymes responsible for modifying this site may offer a route to selective killing of TR‐expressing cells in vivo, even if late stage leukemic cells are predicted to become refractory.

Using TRK99R as a readily detected surrogate for RUNX1 SLGA, we showed that the prototypic t(12;21) cell line REH is refractory, while primary fibroblasts and a primary B‐lymphoblastoid cell line are sensitive. We have shown previously that RUNX1‐mediated SLGA requires integrity of the *CDKN2A* locus in human fibroblasts.^24^ TRK99R induces p16^CDKN2A^ expression in primary fibroblasts and induces growth arrest in primary EBV‐immortalized lymphocytes that retain functional p16^CDKN2A^ but not in t(12;21) REH leukemia cells that fail to express this protein due to gene deletion affecting both alleles of the *CDKN2A* locus.^42^ These observations provide a rationale for the observation that late stage t(12;21) cells express high levels of RUNX1 from the untranslocated allele^22^ and display frequent acquisition of extra copies of chromosome 21 in progressing leukemias.^23^

A significant proportion of t(12;21) leukemias also display loss of p16^CDKN2A^ due to deletion mutations^23^, ^43^ and/or hypermethylation.^44^ Moreover, as RUNX1 SLGA also requires an intact p53 pathway^24^ it is conceivable that some t(12;21) leukemias acquire resistance to RUNX1 SLGA by mechanisms other than loss of p16^CDKN2A^. In further support of the importance of both the p53 and Rb arms of the tumor suppressor response in vivo, the weak oncogenicity of TR in mice is facilitated in *Cdkn2a/Arf* deficient cells.^45^

At the outset of this study, RE presented a paradox as it retains the ability to induce a potent SLGA in fibroblasts.^24^ This observation ran counter to the hypothesis that RE may function as an oncogene by blocking RUNX1‐driven failsafe devices through transcriptional repression of p14^Arf^ expression.^46^ In contrast, we showed that RE induces a potent p53‐dependent SLGA in human primary fibroblasts that lack detectable p14^Arf^ expression, indicating that RE activates p53 by an alternative pathway, with the induction of ROS and MAPK signalling as essential players. Moreover, RE induces p16^CDKN2A^ expression, albeit more weakly than RUNX1.^24^ The notion that RE is oncogenic as a result of transcriptional repression of failsafe pathways is not tenable in light of these observations.

More recent studies have revealed that cellular senescence is not necessarily tumor protective. In the SASP response, senescent cells may release factors that influence bystander cells in a manner that promotes tumor development.^6^ Cytokine release may be beneficial through attraction of immune effector cells and this potential has been explored in clinical trials of cytokine‐based therapies.^47^ However, the other side of the coin is that the SASP includes pro‐oncogenic factors and it is notable that cytokines with an annotated pro‐oncogenic role are among the most prominent in the RE SASP. The ability of RE to promote escape from senescence and immortalization in primary murine fibroblasts is a further indication that RE SLGA is not irreversible, and strongly implicates the SASP in the escape process. Our findings in primary fibroblasts mirror previous studies in CD34+ cells where RE expressing cells displayed an early growth suppression followed by increased self‐renewal, while the surviving cells displayed down‐regulation of DNA repair genes.^48^, ^49^ It would interesting in future to test whether a similar RE SASP is expressed in this context.

It is also interesting to note that the RE SLGA is markedly attenuated in RE9a, a spliced variant of RE that displays significantly enhanced leukemogenic potency in retroviral transduction/transplantation mouse models compared to the full‐length RE product.^26^ The difference was most marked in Hs68 cells where, unlike RE, RE9a induced no obvious morphological change and failed to induce ROS or other markers of senescence. Despite this lack of obvious phenotype, RE9a generates a partial SASP response that may contribute to its ability to promote MEF growth and immortalization and, by extrapolation, its leukemogenicity in vivo. It is important to note, however, that RE9a is not expressed in isolation in t(8;21) leukemia cells, but alongside RE and the unmutated RUNX1 allele, and all three are likely to contribute to regulation of the SASP in vivo.

This study re‐emphasises the heterogeneity of SLGA, despite the common features of growth arrest, flattened morphology, and SA‐β‐gal staining. Subtle differences between RE and RUNX1 SLGA were already evident from morphology and the greater tendency of growth arrested Hs68 cells expressing RE to accumulate in G2.^24^ This study reveals an even more striking difference, as RE induces a complex SASP but RUNX1 either fails to induce or even suppresses production of many of the same mediators. A similar phenomenon was observed for the cell cycle regulator p16^CDKN2A^ which has also been reported to induce senescence without a SASP response.^50^ As p16^CDKN2A^ is both strongly induced by RUNX1 and essential for RUNX1 SLGA^24^ a potential causal link must be considered. Also, from its limited SASP, it may be predicted that ectopic RUNX1 will not trigger extensive inflammatory or innate immune responses in vivo.

The contrasting routes of escape from SLGA in the TR and RE fusions also shed light on the collaborating mutations that are selected in the development and progression of t(12;21) and t(8;21) leukemias. Over 40% of t(12;21) leukemias carry deletions in genes affecting B‐lineage maturation as well as G1/S cell cycle progression checkpoint genes such as *CDKN2A*,^51^ while in t(8;21) leukemias drivers of proliferation predominate, with more than 50% of tumors harboring KIT, NRAS, KRAS, FLT3‐ITD, TKD, CBL, or JAK2 mutations.^52^ Finally, as leukemia and lymphoma cells display resistance to RUNX SLGA, and may even become addicted to RUNX,^13^, ^21^, ^53^ it is possible that drugs directly targeting RUNX‐CBFB functions may have a role to play in future therapeutic strategies.^54^

## CONFLICTS OF INTEREST

The authors disclose no potential conflicts of interest.
